# Pharmacological Mechanisms of Cortical Enhancement Induced by the Repetitive Pairing of Visual/Cholinergic Stimulation

**DOI:** 10.1371/journal.pone.0141663

**Published:** 2015-10-29

**Authors:** Jun-Il Kang, Frédéric Huppé-Gourgues, Elvire Vaucher

**Affiliations:** 1 École d’optométrie, Université de Montréal, CP 6128 succursale centre-ville, Montréal, Qc, H3C 3J7, Canada; 2 Département de Neuroscience, Université de Montréal, CP 6128 succursale centre-ville, Montréal, Qc, H3C 3J7, Canada; University of Alberta, CANADA

## Abstract

Repetitive visual training paired with electrical activation of cholinergic projections to the primary visual cortex (V1) induces long-term enhancement of cortical processing in response to the visual training stimulus. To better determine the receptor subtypes mediating this effect the selective pharmacological blockade of V1 nicotinic (nAChR), M1 and M2 muscarinic (mAChR) or GABAergic A (GABA_A_R) receptors was performed during the training session and visual evoked potentials (VEPs) were recorded before and after training. The training session consisted of the exposure of awake, adult rats to an orientation-specific 0.12 CPD grating paired with an electrical stimulation of the basal forebrain for a duration of 1 week for 10 minutes per day. Pharmacological agents were infused intracortically during this period. The post-training VEP amplitude was significantly increased compared to the pre-training values for the trained spatial frequency and to adjacent spatial frequencies up to 0.3 CPD, suggesting a long-term increase of V1 sensitivity. This increase was totally blocked by the nAChR antagonist as well as by an M2 mAChR subtype and GABA_A_R antagonist. Moreover, administration of the M2 mAChR antagonist also significantly decreased the amplitude of the control VEPs, suggesting a suppressive effect on cortical responsiveness. However, the M1 mAChR antagonist blocked the increase of the VEP amplitude only for the high spatial frequency (0.3 CPD), suggesting that M1 role was limited to the spread of the enhancement effect to a higher spatial frequency. More generally, all the drugs used did block the VEP increase at 0.3 CPD. Further, use of each of the aforementioned receptor antagonists blocked training-induced changes in gamma and beta band oscillations. These findings demonstrate that visual training coupled with cholinergic stimulation improved perceptual sensitivity by enhancing cortical responsiveness in V1. This enhancement is mainly mediated by nAChRs, M2 mAChRs and GABA_A_Rs. The M1 mAChR subtype appears to be involved in spreading the enhancement of V1 cortical responsiveness to adjacent neurons.

## Introduction

Cholinergic fibers projecting from the basal forebrain to the primary visual cortex (V1) modulate the integration of visual stimuli. As the first cortical step of visual processing, V1 is decisive in selecting specific stimuli for transmission to higher cognitive cortical areas. Cholinergic modulation of V1 thus results in strong effects on the fine-tuning of conscious visual perception. In previous studies, we showed that the repetitive coupling of visual stimulation with cholinergic stimulation could provide strong and long-term changes in the visual capacity of rats [1–4]. Repetition is particularly important because it can consolidate neural pathways and increase the neural efficiency of perceptual processing, especially when coupled to cholinergic stimulation [5–7]. Therefore, understanding the neuropharmacological mechanisms of the long-term enhancement of visual responses by acetylcholine (ACh) might aid in the identification of appropriate pharmacological targets for the improvement of visual processing and performance.

The neuronal effects of ACh on V1 are very complex, although quite well described. The neuronal effects of ACh on V1 differ depending on the receptor subtype and location [8–12]. In acute experiments, administration of ACh increases the thalamocortical signal in layer IV of V1 through presynaptic nicotinic cholinergic receptors (nAChR) [8, 13] and the M1 subtype of the muscarinic cholinergic receptor (M1 mAChR) located postsynaptically [14, 15]. Administration of ACh also modulates inhibition by activating GABAergic interneurons [16] through nAChRs [17, 18] and M1 mAChRs [19] and by suppressing GABA release through the M2 subtype of muscarinic cholinergic receptors (M2 mAChR) [19, 20]. This cholinergic influence on the GABAergic system activity is particularly relevant in sensory processing and perceptual learning given the involvement of the GABAergic neurons in oscillations in the gamma range (30–90 Hz) [21] and in connectivity changes [22], two mechanisms related to attention, learning and cortical plasticity. It has also been shown in acute experiments that basal forebrain stimulation [23, 24] or the intracerebral injection of cholinergic agonists [25] produce high frequency oscillations. However, the specific involvement of different mAChR subtypes in these mechanisms is not known because non-selective inhibitors of muscarinic cholinergic receptors (i.e., scopolamine, atropine, etc.) have been used in most of the previous studies. Moreover, the effect of repeated cholinergic activation over long periods of time has not been extensively studied.

Based on the previous data, the present study was designed to investigate the involvement of different cholinergic and GABAergic receptor subtypes in cortical responsiveness after the cholinergic enhancement of visual training. Daily pairing of visual stimulation with basal forebrain electrical stimulation (VS/HDB) was performed over a one week period with the simultaneous intracortical infusion of the following agents: mecamylamine, a non-selective antagonist of nAChR; pirenzepine, a M1 mAChR antagonist; AF-DX116, a M2 mAChR antagonist; picrotoxin, a GABA_A_R antagonist; or muscimol, a GABA_A_R agonist. The effects of these treatments on cortical activity, visual detection thresholds and neuronal synchronization were measured by comparing the visual evoked potential (VEP) responses in V1 to various spatial frequencies before and after VS/HDB training. Cortical visual acuity was extrapolated from the results of VEP recordings elicited by diverse spatial frequencies [26–28]. The results revealed an increase in the cortical response following repetitive VS/HDB stimulation that was mediated by nAChRs and cortical microcircuit disinhibition via M2 mAChRs. Moreover, time-frequency analyses revealed an increase in neuronal synchronization in the beta and gamma frequency bands following VS/HDB training.

## Materials and Methods

### Animal preparation

Adult Long-Evans male rats (n = 63, 200–225 g) were obtained from Charles River Canada (St-Constant, Quebec, Canada) and were maintained in a 12-h light/dark normal daylight cycle with *ad libitum* access to food and water. The guidelines set by the Canadian Council for the Protection of Animals were followed for all procedures and approved by the local Animal Care Committee, “Comité de Déontologie de l’Expérimentation sur les Animaux” at the Université de Montréal (protocol # 12–172). All efforts were made to minimize suffering and the number of animals used for these experiments.

### Experimental design

**Fig 1:**
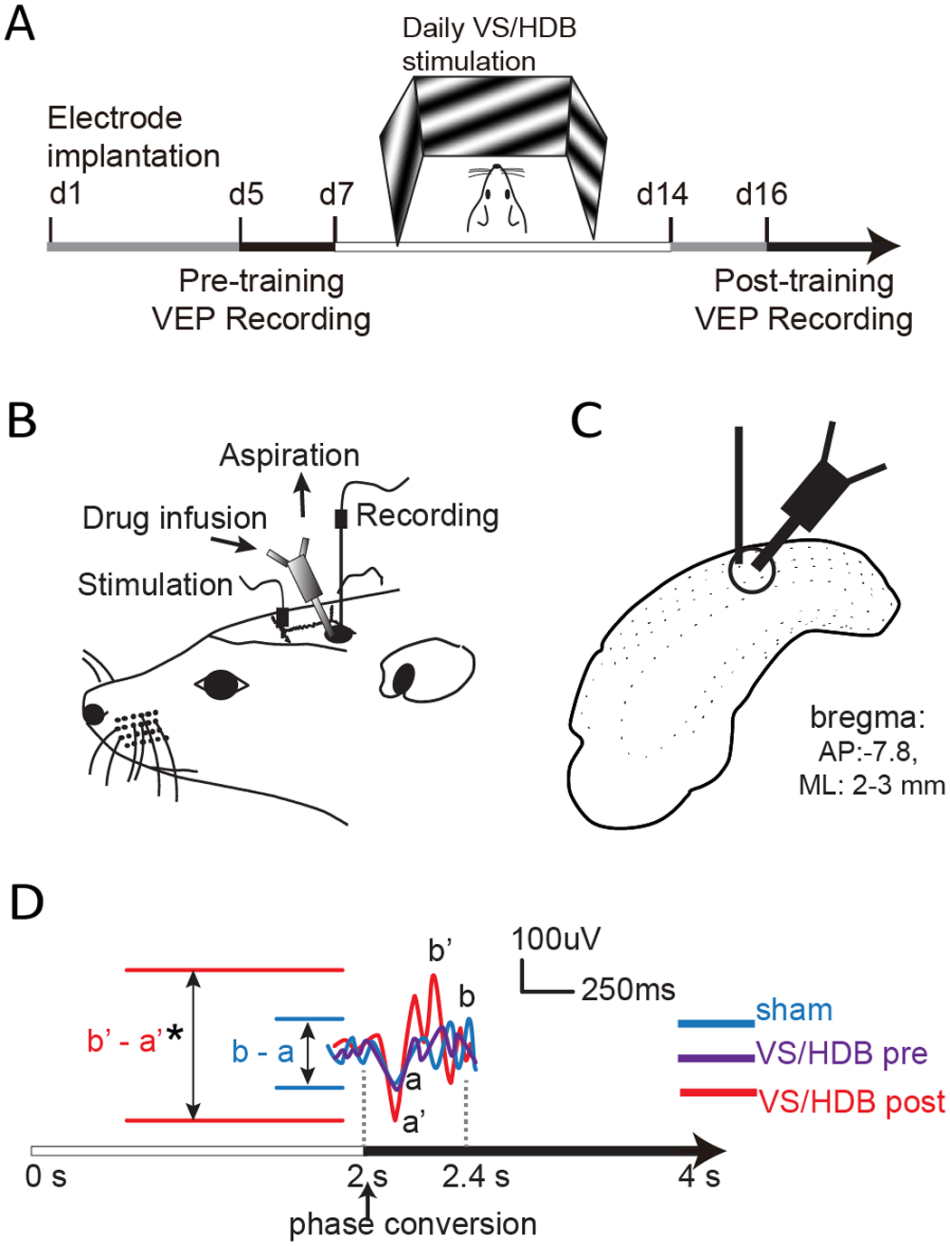
Design of the experimental procedure. A) Timeline of the different experimental steps. The pre-training visual cortical responses to visual stimulation were recorded 4 days (d5) after the implantation of the electrodes and guide cannulas. Visual training was provided for 10 min/day for 7 days (d7-d14) and followed by the recording of the post-training VEPs (d16) (see text for details). B) Schematic diagram illustrating the chronic implantation of the recording electrode and the push-pull guide cannula in V1. The stimulating electrode was implanted in the HDB. C) Schematic representation of the areas of pharmacological agent injection and electrophysiological recording. D) Representative VEP signal traces in response to a 0.12 CPD grating for the sham, pre- and post-training VS/HDB groups. The VEP was evoked by phase inversion after 2 seconds of stimulus presentation (0.25 Hz), and the amplitude was measured by subtracting the negative peak (a or a’) from the positive peak (b or b’). Note that the visual cortical response increased after the VS/HDB training (b’-a’) compared to sham (b-a).

Recording electrode and injection guide were implanted in and over the rat V1, respectively, prior to VEP recording (day 1). Pre-training VEPs were recorded (day 5) followed by 7 days of VS/HDB training (day 7–14). Post-training VEPs were then recorded (day 16) (Fig 1A). Then, rats were euthanized with an overdose of pentobarbital and perfused with paraformaldehyde 4% in 0.1 M phosphate buffer, pH 7.4.

### Implantation surgery

To implant stimulating (basal forebrain) and recording (V1) electrodes and cannula guides, animals were anesthetized with isoflurane (induction 5%, maintain 3%) and placed in a stereotaxic apparatus. Throughout the experiment, the rectal temperature was maintained at 37°C using a thermostatically controlled heating pad (FHC, Bowdoinham, ME, USA). A dental drill was used to make 2 ipsilateral holes in the skull, one above the left visual cortex and one above the horizontal limb of the diagonal band of Broca (HDB). The electrode guide (polyurethane tubing) was placed above V1 (mm from Bregma: AP -7.5, ML +4.0, DV 0) [29] and a push-pull cannula guide (Plastics1, Roanoke, VA) was inserted adjacent to the electrode guide (from Bregma: AP -7.5, ML +3.6, DV -0.7 mm, 30° angle from verticality). A tungsten-stimulating electrode denuded at each end was implanted in the HDB ipsilateral to the cortical recording site (mm from Bregma: AP -0.3, L +2.0, DV -9.0). The guides and the HDB implanted electrode were secured with dental cement, and two stainless steel screws (Small parts, Miami Lakes, FL, USA) were installed at the skull surface to hold the dental cement (Fig 1B). After suturing the incised skin, local anesthesia (xylocaine 2%, Astra Zeneca, Mississauga, Canada) was topically administered to the wound, and the animals were returned to their cages. An anti-inflammation agent, carprofen (Rimadyl, 5 mg/kg s.c.), was administered to the animal for prophylaxis. The recording site was identified by an electrolytic lesion made after the last VEP recording and then the electrode placement was confirmed by Cresyl violet staining of the fixed brain sections using a Leica DMR microscope and the rat brain atlas [30]. This observation confirmed that the stimulating electrode was implanted in HDB and recording electrode was implanted in V1, respectively. We also verified by injecting Chicago sky blue (1% in saline) through the push-pull cannula prior to the terminal PFA fixation of the rat, that the recording electrode tip was within the volume of vehicle infusion in V1 (Fig 1C).

### VEP recording procedure

The LFP recording method was chosen to observe cortical modifications [2, 27]. The VEP was elicited by contrast reversal of sinusoidal grating patterns [28] and recorded ipsilateral to sites of HDB stimulation, as previously described [2, 3]. For VEP recordings, the animals were anesthetized (isoflurane, induction 5%, maintenance 1.5%), placed in a stereotaxic apparatus and then kept in the dark. The electrode guide was removed, and the recording tungsten electrode (FHC, < 1Mohm) was inserted 0.5 mm below the dura. Visual stimuli were displayed on a computer monitor that was placed 30 cm at the right side parallel to the animal’s midline (left eye closed) and centered on the eye. As described previously [2], visual stimulation consisted of an oriented sine-wave grating with a 90% contrast and phase inverting at 0.25 Hz or a baseline control gray screen of 0 cycles per degree (CPD) [28]. The electrical signal was recorded for 1500 ms. A 0.25 Hz phase inverting frequency was chosen to avoid overlap responses between VEPs. To avoid an orientation preference bias—due to a preferred response of the cortex to a specific orientation—and to verify that the cortical enhancement could be elicited by any trained orientation, we recorded in distinct animals the VEP for 3 different orientations, called collectively X° [26, 27]. Orientations that were shown to evoke a weak response (i.e., 30°, 45° and 60°, called suboptimal orientation) [31] were selected as X° to avoid a ceiling effect (no improvement of visual acuity possible). A ceiling effect has been previously shown for some optimal orientation (0°: horizontal and 90°: vertical) [3]. The same animals were also tested (but not trained) for a X+90° orientation to test the orientation selectivity of the training effect. During VEP recording, nine different spatial frequencies (0, 0.08, 0.12, 0.3, 0.5, 0.7, 0.8, 0.9, 1.0 CPD) of X° and X+90° orientations were presented in a pseudo-random manner. The corresponding orientation X°, was used during the visual training (7 days of visual/cholinergic stimulation pairing) and recording of VEPs. Evoked responses were amplified (5,000X), filtered at 3 Hz ~ 1 kHz (Grass Inc., West Warwick, RI, USA) and collected with the MP100 data acquisition system and Acqknowledge software (v 3.8; Biopac system Inc., Goleta, CA, USA).

### VEP analysis

The mean amplitude of the VEPs was calculated by measuring the electrical responses of extracellular field potentials elicited by the visual stimuli presentation (contrast reversal). The signal amplitude was obtained by measuring the difference between the negative peak and the positive peak of the VEPs or the baseline. Each analysis was performed between 0–400 ms after the contrast reversal and was averaged for each orientation and spatial frequency. Baseline was considered to be the mean response of 20 averaged cortical responses that were measured while showing test subjects a consecutive series of only grey screens. The results were analyzed by a blind experimenter. The difference between pre-pairing VEP and post-pairing VEP was used as a measure of cortical activation (Post—PreVEP, Fig 1D).

### Time-frequency analysis

To examine the evolution of VEP phases over time, a short-time Fourier transform (STFT) spectrogram function was used in Matlab (Mathworks, Nattick, MA, USA) [32]. The function ‘[S,F,T,P] = spectrogram (DATA)’ returns each value in variables (where S is the STFT coefficient, F is the analyzed frequency, T is the time, and P is the power spectral density) after performing Fourier transform analysis on DATA (i.e., VEP). The functional equation was provided by Mathworks (http://www.mathworks.com/help/signal/ref/spectrogram.html). The power spectral density (PSD) matrix during the (i, j) entry, i.e., the PSD value for a given frequency with bin i and time frame j, was obtained by *P*(*i*, *j*) = *k*|*S*(*i*, *j*)|^2^, where k is a real-valued scalar defined as

$$k=\frac{1}{Fs\sum_{n=1}^{L}{\left|w\left(n\right)\right|}^{2}}$$

where w(n) is 200, a hamming window with 100 overlapping samples. Fs is the sampling frequency, which was 2000 samples/s of signal in this study. S(i, j) is the STFT coefficient of the discrete signal s[n]. L denotes the length of the analysis window, w(n), used during the STFT. The frequency resolution and time resolution were 1 Hz and 50 ms, respectively. The PSD (i.e., the values of variable P) between 30–90 Hz, which corresponds to gamma band oscillations, and 15–30 Hz, corresponding to beta band oscillations, were summed separately, and the expression was analyzed over 500 ms with a time window of 50 ms. Because the visual stimulus changes every 2 s (Fig 1D), it is not possible to accurately compare lower frequency power. Hence, only high frequencies (>7 Hz) of PSD were analyzed. PSDs were collected only for the results that evoked VEP amplitudes that were three-fold bigger than the standard deviation of the baseline amplitude. Other results were considered to be failed attempts to discriminate the contrast reversal and were excluded from collection.

### Drug infusion

All drugs were obtained from Sigma Chemical Company and were dissolved in a saline solution. Muscimol (GABA agonist: 200 μM), Picrotoxin (GABA antagonist: 100 μM) [33], Pirenzepine (M1 mAChR antagonist: 100 μM) [34], AF-DX 116 (M2 mAChR antagonist: 8 nM) [35], mecamylamine (nAChR antagonist: 10 μM) [2] or vehicle (saline) were administered using an injection pump (PHD, Harvard Apparatus, Holliston, MA, USA). The push-pull cannula allowed for excess fluids at the injection site to be discarded and limited the accumulation of the drug within the cortex (Fig 1B).

### Repetitive visual/cholinergic stimulation pairing

The visual training paradigm was designed to examine whether the selective orientation response could be modified through the visual training of a specific pattern and/or through cholinergic neuron stimulation. The stimulus was either a gray screen for the control group or a sine-wave grating (0.12 cycle/degree, orientation X°, phase inverting at 1 Hz) for other groups. Depending on the pharmacological agent injected during visual training, rats were divided into seven groups (Table 1). During daily training, the animals were restrained for 10 min a day for 7 days. The animals were awake with their heads fixed in a frame surrounded by three monitors (2 laterals and 1 frontal) placed 21 cm away from their eyes (Fig 1A). The visual stimulus was generated using VPixx software (v 2.79, VPixx technologies Inc., Saint-Bruno, Quebec, Canada) and displayed on the three monitors (LG, luminance 37 cd/m2). Training was performed daily for each rat at the same time of the morning while infusing drugs through the push-pull cannula.

**Table 1:** Experimental groups.

Name	Treatment	N
**CTL**	sham exposure/no HDB stimulation/saline injection	6
**VS**	X° sine-wave grating presentation/no HDB stimulation/saline injection	6
**VS/HDB**	visual exposure/HDB stimulation/saline injection	6
**VS/HDB/PTX**	visual exposure/HDB stimulation/picrotoxin injection	8
**VS/HDB/muscimol**	visual exposure/HDB stimulation/muscimol injection	9
**VS/HDB/PZP**	visual exposure/HDB stimulation/pirenzepine injection	6
**VS/HDB/AFDX**	visual exposure/HDB stimulation/AFDX-116 injection	7
**VS/HDB/MEC**	visual exposure/HDB stimulation/mecamylamine injection	4
**VS/muscimol**	visual exposure/muscimol injection	5
**VS/AFDX**	visual exposure/AFDX-116 injection	6

### HDB electrical stimulation

Electrical stimulation started at the beginning of the visual stimulation period and was delivered for 10 min (train of pulses 100 Hz, 0.5 ms, 50 μA, 1 sec on/1 sec off, Pulsemaster A300, WPI, Sarasota, FL) through a current source (WPI 365, WPI, Sarasota, FL). This paradigm of electrical stimulation in the HDB is known to activate the cholinergic system preferentially in comparison to the GABAergic system [3, 36].

### Statistical analysis

The visual cortical response threshold was determined in the sham group by comparing the amplitude of VEPs against different spatial frequency sinusoidal gratings and the grey screen using a paired t-test. The difference in the amplitude of the VEPs (Post—PreVEP, Fig 1D) and the PSD of time-frequency analysis between the sham, VS and VS/HDB groups (Table 1) were tested by one-way ANOVA followed by Tukey post-hoc analysis. Pharmacological effects within the VS/HDB group (i.e., VS/HDB, VS/HDB/MUS, VS/HDB/PZP/ VS/HDB/AFDX, VS/HDB/MEC/ and VS/HSB/PTX) and within the VS group (i.e., VS, VS/MUS and VS/AFDX) were compared on the Post—PreVEP value using one-way ANOVA and the Dunnett post-hoc test (compared with VS/HDB or VS, respectively). All statistical analyses were carried out with SPSS 19.0 for Windows 7 (SPSS Inc., Chicago, IL, USA) at a significance level of p < 0.05.

## Results

### Increase in the VEP amplitude following VS/HDB stimulation pairing

**Fig 2:**
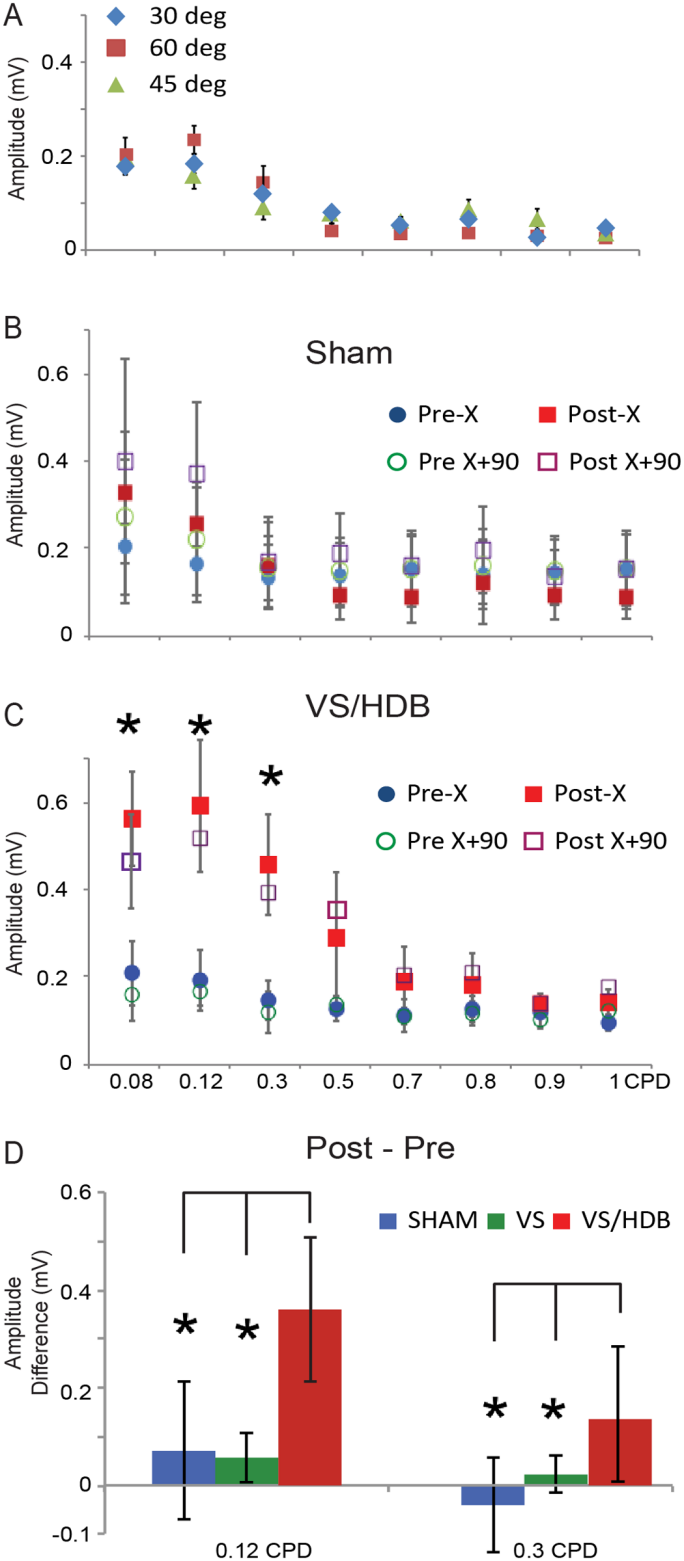
Effects of repetitive Visual/cholinergic stimulation (VS/HDB) on VEP amplitudes. A) Basal VEPs in response to 30°, 45° and 60° stimuli orientation recorded prior to any experimental procedure. There were no differences in VEP amplitudes between the orientations, which were subsequently pooled into the X° and X+90° groups. B) VEP amplitudes from the sham (grey screen/no HDB stimulation) animals in response to different orientations and spatial frequencies. There were no significant differences between the pre- and post-training values. C) VEP amplitudes in the repetitive VS/HDB stimulation (training) animals in response to different orientations and spatial frequencies. Visual/cholinergic training induced increases in VEP amplitudes in response to the exposure of the stimulus (0.12 CPD) and higher spatial frequency stimuli (0.3 CPD). D) VEP amplitude difference (post training—pre training) for X°-0.12 CPD (left) and 0.3 CPD (right). VEP difference of VS/HDB was significantly enhanced compared to sham and VS group. (*, ANOVA, post-hoc Tukey, p < 0.05). VS/HDB = sinusoidal grating screen with HDB stimulation, VS = sinusoidal grating screen without HDB stimulation, and Sham = grey screen without HDB stimulation. The error bars represent the average deviation.

The VEP response and amplitude were similar across all orientations tested (30°, 45°, and 60°, collectively termed "X°") (Fig 2A), as well as across all X+90° stimuli (i.e., 120°, 135° and 150°; data not shown). These values were thus further pooled together for the X° and X+90° analysis. The VEP responses in the pre-training/sham recordings were largest for 0.08 and 0.12 CPD both for the X° and X+90° stimuli (Fig 2B). The threshold of detection of the contrast inversion was 0.5 CPD because cortical responses were not significantly different from the baseline level (field potential during grey stimulus presentation) above this spatial frequency in the sham group (grey screen: 0.03 ± 0.019 mV v. 0.5 CPD: 0.25 ± 0.14; paired t-test, p = 0.081; average ± average deviation). Thus, 0.5 CPD was determined to be the cortical visual acuity in these experiments.

To compare the VEP change between all groups, we calculated the difference of VEP amplitude by subtracting the pre-pairing VEP from the post-pairing VEP (Post—PreVEP: see Methods). There were no post- versus pre-pairing changes in the VEP amplitude in the sham group or visual stimulation only (VS) group for any of the spatial frequencies studied (0.07±0.14 mV and 0.05±0.05 mV, respectively for 0.12 CPD, Fig 2D). There was a significant increase of the Post-Pre VEP value to X° in the repetitive VS/HDB stimulation group (VS/HDB) compared to sham group (post—pre at 0.12 CPD: 0.36 ± 0.15 mV, F[2, 19] = 8.825, one-way ANOVA, post-hoc Tukey, p = 0.008; Fig 2D) or VS group (p = 0.003). The increase in VEP amplitude in the VS/HDB group compared to the VS group was observed for the trained spatial frequency (0.12 CPD) but also for higher spatial frequencies up to 0.3 CPD (Fig 2C, one-way ANOVA, p = 0.007 and p = 0.002 compared to sham and VS, respectively). These results are indicative of an increase in V1 responses to the exposed stimulus and a transfer of this enhancement to adjacent higher spatial frequencies following repetitive VS/HDB stimulation. However, the visual acuity threshold was not changed because there was no augmentation above 0.3 CPD.

The VS/HDB stimulation effect was not transferred to other orientations (i.e., X+90°) because there was no significant change of X+90° Post-Pre VEP value compared to the sham counterpart (p = 0.054 for 0.12 CPD and p = 0.152 for 0.3 CPD; Fig 2C). This signifies that the VS/HDB pairing induces an orientation specific augmentation of a cortical response for trained and higher spatial frequencies but not above the visual acuity threshold.

### Involvement of the nAChR, mAChR and GABAergic systems

To evaluate the different pharmacological players in the enhancement effect of VS/HDB stimulation in V1, we locally administered different agonists and antagonists of the cholinergic and GABAergic receptors into V1 during each VS/HDB stimulation period. The VEPs were recorded without any drug administration.

**Fig 3:**
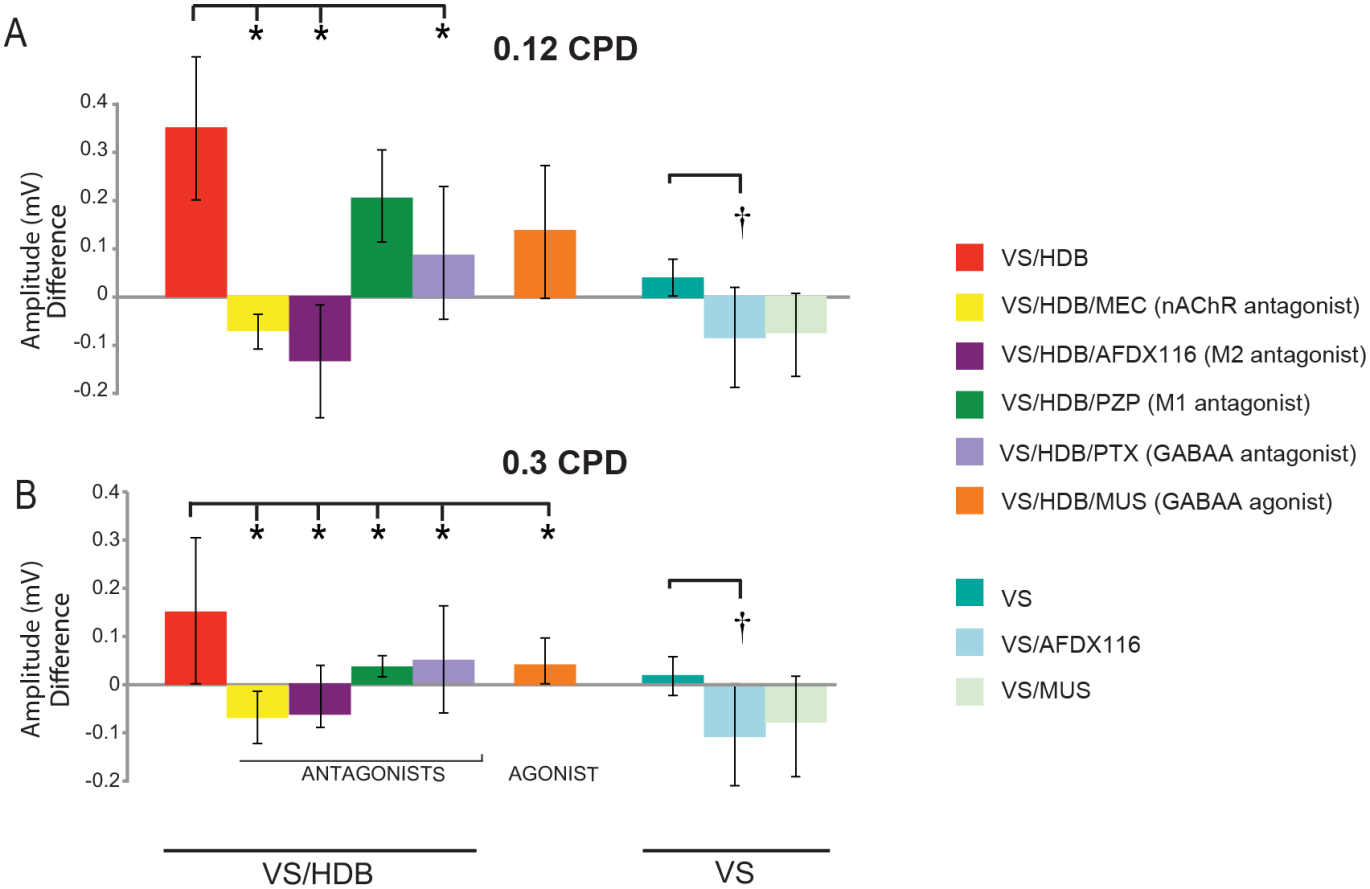
Change in VEP amplitudes following pharmacological modulation during visual/cholinergic stimulation (VS/HDB). The histograms represent the VEP difference of amplitude (Post-Pre training) for the different groups for the trained spatial frequency of 0.12 CPD (A) and 0.3 CPD (B). Note that the VEP amplitude enhancement following the visual/cholinergic training was blocked by MEC, AFDX, or PTX injection. (*; one-way ANOVA, post-hoc Dunnett, p < 0.05, compared to VS/HDB group, †; post-hoc Dunnett, p <0.05, compared to VS group). The error bars represent the average deviation. (Abbreviations; PZP: pirenzepine; MEC: mecamylamine; AFDX: AF-DX 116; MUS: muscimol and PTX: picrotoxin).

Blockade of nAChRs (mecamylamine: VS/HDB/MEC) disrupted the VEP amplitude enhancement induced by VS/HDB pairing for the trained 0.12 CPD spatial frequency (F [5,39] = 7.014, ANOVA, post-hoc Dunnett, p = 0.001 compared to VS/HDB) (Fig 3A). Antagonism of the M2 mAChR (AF-DX116) not only disrupted the VEP amplitude enhancement for the trained spatial frequency (p < 0.001) but also appeared to reduce the VEP amplitude compared to the basal level. Comparatively, specific blockade of M1 mAChR with pirenzepine did not disrupt the enhancement effect induced by VS/HDB pairing at 0.12 CPD (Fig 3A, p = 0.414).

We further injected GABAergic agents to examine whether GABAergic neurons were involved in the VEP enhancement induced by repetitive VS/HDB pairing. GABA_A_R antagonism via picrotoxin injection (VS/HDB/PTX) disrupted the enhancement effect after the VS/HDB pairing (Fig 3A, p = 0.025). In contrast, muscimol (a GABA_A_R agonist, VS/HDB/MUS group) injection during each VS/HDB stimulation period did not change the VEP enhancement from VS/HDB treatment (p = 0.075, Fig 3A). These signify that the activation of GABA_A_R, nAChR and M2 mAChR mainly mediate VEP enhancement in response to stimulus training.

The transfer of VEP enhancement induced by VS/HDB to higher spatial frequencies (up to 0.3 CPD) was more broadly altered by drug injections (Fig 3B). Every pharmacological substance injected during VS/HDB pairing significantly disrupted the VEP enhancement effect for 0.3 CPD (p < 0.001, P < 0.001, p = 0.003, p = 0.001, p = 0.003 for VS/HDB/MEC, VS/HDB/AFDX, VS/HDB/PZP, VS/HDB/MUS, VS/HDB/PTX, respectively). These results suggest that the transfer of VEP enhancement mechanism involved all of the receptors tested (i.e., nAChR, M2 mAChR, M1 mAChR and GABA_A_R).

To clarify the VEP amplitude decrease after M2 mAChR antagonist (AFDX-116) injection and to determine the role of the GABAergic drive in this response, additional groups were tested with only visual stimulation and no HDB stimulation. The VEP amplitudes of the GABAergic agonist-injected group (VS/Mus) were not changed compared to the VS group (F [2,18] = 4.317, one-way ANOVA, post-hoc Dunnett, p = 0.163, compared to VS), but blocking the M2 mAChR (VS/AFDX) significantly reduced the VEP amplitude (p = 0.024, Fig 3A and 3B). These results indicate that GABAergic activation alone does not induce a long-term cortical modulation but that M2 mAChR antagonism during visual stimulation could suppress the cortical response in a manner similar to long-term depression.

### Gamma and beta band cortical oscillations increase following repetitive VS/HDB stimulation

**Fig 4:**
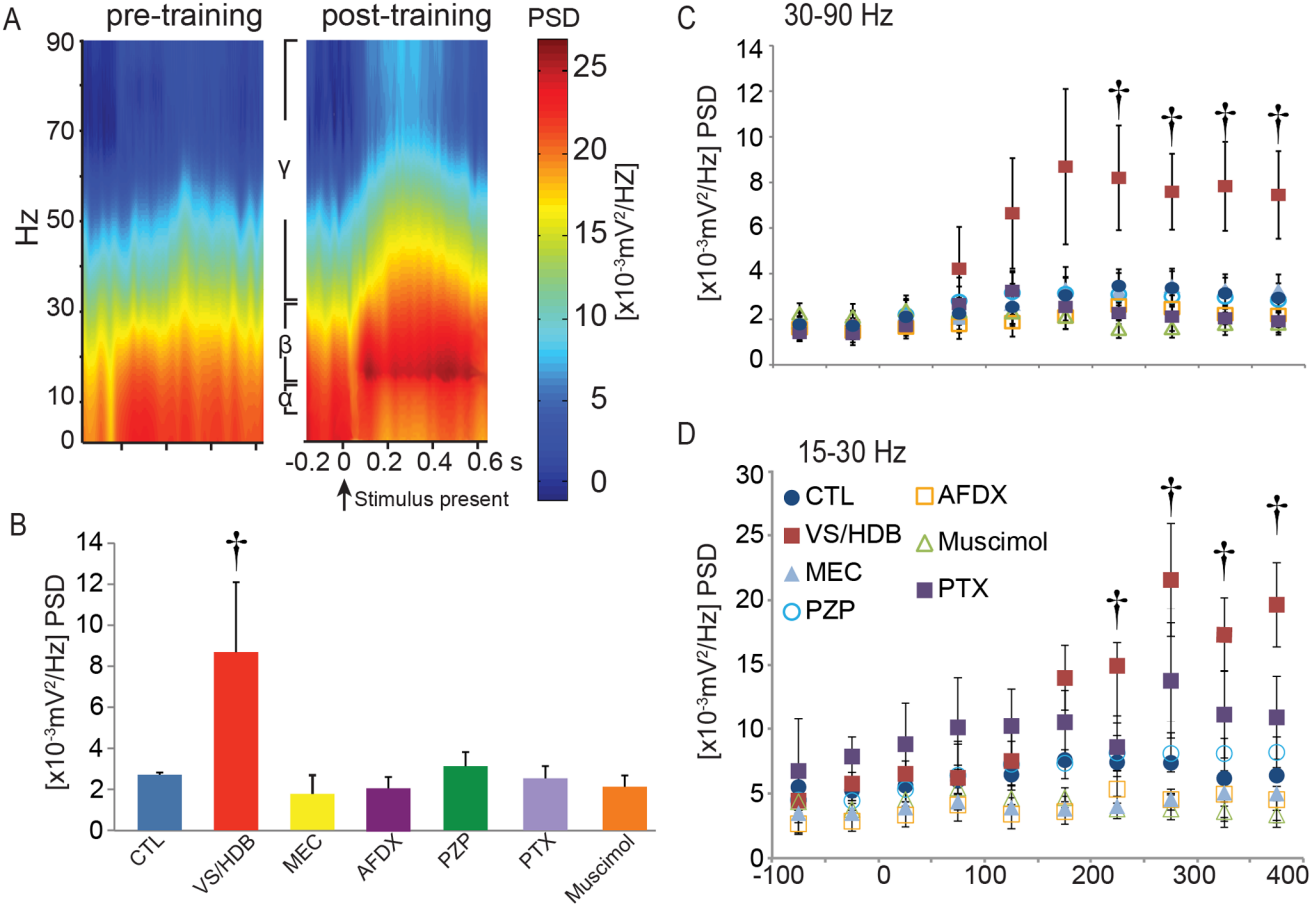
Time-frequency VEP analyzes. A) Representative comparison of the power spectral densities (PSDs) of the pre- and post-training VEPs from the VS/HDB group as analyzed with the short-time Fourier transformation. B) Comparison of the PSDs in the 200–250 ms time window after the stimulus presentation. Only the VS/HDB group exhibited an increase in gamma band oscillations (†, one-way ANOVA, p < 0.05). Gamma (C) and beta band (D) power during VEP recording. There was an increase of beta (15–30 Hz) and gamma band (30–90 Hz) power 200–250 ms after stimulus onset.

To evaluate the frequency changes in the cortical response to visual stimulation, we performed a time-frequency analysis utilizing a short-time Fourier transformation of the VEP results (50 ms time window) and compared the power spectral densities (PSDs). Because significant differences in the PSDs were not observed across the spatial frequencies (data not shown), the data were pooled together. The spectral analyses of the VEP results revealed that 2 days after the repetitive VS/HDB stimulation, the neuronal activation in the beta band frequency began to increase at 200 ms after stimulus onset (one-way ANOVA, F [2, 19] = 15.024, post-hoc Tukey, p = 0.002 compared to sham) and the gamma band frequency increased after 200 ms (ANOVA, F [2, 19] = 5.882, p = 0.044) (Fig 4A). These increases remained significantly different until 400 ms after stimulus presentation (Fig 4C and 4D). This effect was abolished by each of the drugs, which is suggestive of a combined action of ACh and GABA (Fig 4B). Comparatively, we did not observe any significant changes in the alpha frequency band. These results suggest that the enhancement of the cortical response after VS/HDB pairing was correlated with an increase of neuronal synchronization in high frequency oscillations and indicate that disruptions in either the cholinergic or GABAergic system can alter these oscillations.

## Discussion

Our results revealed that repetitive VS/HDB stimulation induced an increase in the VEP amplitude that was sustained across subsequent visual stimulations with the trained stimulus. This increased amplitude was spread to higher spatial frequencies than the trained one, although the visual acuity of the rats, as measured by electrical recordings, was not changed. The nAChR, M2 mAChR or GABA_A_R antagonists used herein prevented VEP potentiation induced by VS/HDB. The M2 mAChR antagonist might, however, exert a general suppressing effect because it also decreased the basal cortical responses compared to control levels in the VS condition. Interestingly, M1 mAChR antagonists and GABA_A_R agonists only blocked the transfer of VS/HDB induced VEP enhancement for untrained stimuli. Together, these results suggest that pairing visual stimulation with HDB stimulation can boost the V1 response to subsequent visual stimuli. Further, activation of the GABA_A_R alone does not appear to induce cortical plasticity; however, M2 mAChRs appear to be involved in the inhibitory control of cortical microcircuitry.

### Repetitive VS/HDB stimulation increases the long-term response to the trained stimulus

As expected, repetitive VS/HDB stimulation induced a long-term increase in the V1 neuronal response to subsequent trained visual stimuli, observed when the VEPs were recorded 2 days after the last VS/HDB stimulation or drug administration. The electrical stimulation used in this study was developed [36] and shown [2, 3] to favor cholinergic system activation. Although we cannot exclude the possibility that GABAergic activation is implicated, many studies support that the cholinergic system is the main factor during VS/HDB stimulation. Studies have also shown that pairing cholinergic and sensory stimulation can induce perceptual learning [3, 5, 23, 37]. In agreement with these findings, the data herein suggest that repetitive VS/HDB stimulation improves the long-term ability to detect contrast changes of visual stimuli via cholinergic system.

The repetitive VS/HDB stimulation induced also a long-term increase in the synchronized neuronal activity observed by gamma and beta frequency band oscillations. This finding is in agreement with a role of VS/HDB enhancement in perceptual learning because it has been suggested that oscillations in the gamma frequency range reflects cognitive activities, such as the processing of sensory input [21] or attention and learning [38]. Comparatively, beta band oscillations are found mostly during sensorimotor processes [39, 40], although they are also increased during top-down attentional processes [41–43]. Previous studies have also demonstrated that acute cholinergic stimulation can increase beta band [44] and gamma band activities [25, 45] in a manner that correlates with the enhancement of visual encoding [24] or contrast sensitivity [23]. The present finding suggests that VS/HDB pairing promotes the long-term synchronization of neuronal activities for a specific stimulus and amplifies visual information during attentional processes.

The increase in the electrophysiological response induced by VS/HDB training might reflect either an increase of responsiveness of specific neurons, an increased number of responsive neurons or an increased number of synchronized neurons during visual processing [46, 47]. First, as it was shown that stimulation during a neuronal oscillation phase peak could induce LTP [48–50], improved neuronal synchronization migth have contributed to increased long-term VEP amplitude in the VS/HDB group. However, all of the administered drugs altered the synchronization of the neurons, but some drugs (pirenzepine and muscimol) did not alter the potentiation effect at 0.12CPD, suggesting that neuronal synchronization is not the only requirement for the long-term enhancement of visual processing. Second, it is probable that cholinergic stimulation during visual training improves stimulus sensitivity of the neurons [23, 51] and cortical plasticity [28]. The current study is consistent with numerous previous studies demonstrating the implication of the cholinergic system during the enhancement of neuronal responses [2, 13, 23, 52]. Lastly, similar with the increase in the number of responsive neurons after perceptual learning [27], VS/HDB pairing can also increase the responding neurons by reinforcement of thalamocortical pathway [13, 53]. The alteration of training-induced changes of VEPs by the inhibition/activation of receptors suggests plausible pharmacological mechanisms for these aforementioned phenomena.

The nAChRs seem to be a crucial player in the increase in the electrophysiological response induced by VS/HDB training. The nAChRs are primarily found in the presynaptic thalamocortical afferents in layer IV, and they have been shown to strongly facilitate thalamocortical inputs [8, 13, 54]. Although the isoflurane can affect nAChR during VEP recording [55], the mechanisms of VEP enhancement is likely due to nAChR-mediated facilitation of thalamocortical inputs in awaken state. Alternate mechanisms include induction of cortical LTP-like process and increased glutamate release because it has also been shown previously that 1) nAChRs are involved in LTP-like mechanism induced by VS/HDB pairing [2] and 2) that nAChRs regulate glutamate release in the sensory cortex [54, 56, 57]. Moreover, nAChRs might be involved in fine regulation of cortical plasticity because its inhibition releases the plasticity brake, Lynx1, which is crucial for induction of the cortical plasticity and recovery of visual function [28].

The involvement of M2 mAChRs in the potentiation of the cortical response was expected because M2 decreases ACh release [58–60] and decreases GABAergic inhibition [19](see discussion below). The lack of M1 mAChR effects on the potentiation of the cortical response was quite surprising, however, because this receptor represents 40% of the mAChRs in V1 and generally enhances the glutamatergic drive. The lack of prevention of VS/HDB-induced cortical enhancement by M1 antagonist might have resulted for here by a possible M1 inhibitory effect, as exerted in layer V, where M1 mAChR presynaptic localization reduces glutamate conductance [61]. Moreover, in the primate’s visual cortex, M1 mAChRs seem to be largely expressed on GABAergic interneurons [62], where the role of their expression is unclear.

Herein, GABA_A_R stimulation did not change the VS/HDB-induced potentiation of VEPs, which appears to be inconsistent with the general inhibitory role of GABA in the cortex. However, it is possible that GABA_A_R activation is weaker than the involvement of other excitatory receptors, such as nAChRs. For example, it has been shown in hippocampal slice preparations that the whole cell current induced by nAChR activation was increased after GABA release [63]. So, it is possible that ACh excitatory effect during VS alone [64] or VS/HDB did overcome the GABA_A_R activation by muscimol. In contrast, the blockade of GABA_A_R prevented the VS/HDB-induced potentiation effect. This suggests that lateral competition was increased [65] by picrotoxin administration and hence disrupts VEP enhancement, mechanism related to the involvement of GABAergic neurons in reducing the width of the tuning curves of V1 neurons [66, 67]. Alternatively, the GABA_A_R antagonist might also have blocked the effect of the GABAergic basal forebrain projections, although these one are not predominantly activated during electrical stimulation of the HDB [16, 36].

Overall, the cortical modifications that result from VS/HDB training might increase stimulus sensitivity, facilitate discrimination and increase the perceptibility of visual stimuli with mechanisms that are mostly related to nAChRs and GABAergic inhibition.

### Repetitive VS/HDB stimulation enhancement spread to other stimulus attributes

The present results show a spread of the VS/HDB enhancement of VEP amplitude to higher spatial frequencies than trained. These results agree with those of our previous studies showing that repetitive VS/HDB stimulation induces an increase in visual discrimination capacity to higher spatial frequency stimuli measured behaviorally [3, 4]. The improvement in visual discrimination shown in the previous study was correlated with an increase in VEP amplitudes (REF 3; Pearson’s correlation, R = 0.725, p = 0.001). The spread might be supported by an increased number of neurons responding to the visual stimulation [27], i.e., by remodeling the thalamocortical input in V1 [53]. However, no increase in visual acuity (visual detection threshold compared to control experiment) *per se* was detected in the present study. As there is an obvious link between the neural response and behavioral performance [68], it is possible that an increase of visual acuity could have been detected by behavioral testing, reflecting the improvement in the 0.3 CPD response observed here.

The antagonism of M1 mAChRs blocked the effects of VS/HDB training at a higher spatial frequency (0.3 CPD), but not at the trained spatial frequency. This suggests that M1 plays a role in the spread of the enhancing effects of VS/HDB pairing rather than in the amplification of the thalamocortical signal. It is suggested that pre-synaptic M1 mAChRs focus feed-forward inputs by decreasing lateral inhibition (competition) and promote the transmission of visual training effects by increasing the feed-forward response at the post-synaptic level [69]. Compared to nAChRs, which are located principally on presynaptic thalamocortical fibers, M1 mAChRs are widely distributed in V1 [17], and M1 functions vary depending on their location [8]. M1 mAChRs emphasize the feed-forward transmission of a selective stimulus through post-synaptic mechanisms [14] or through the pre-synaptic inhibition of lateral connections that would decrease the lateral spread of thalamocortical inputs [70] and prevent competition between feed-forward excitations. In agreement, the M1 antagonist abolished the synchronization between neurons. Moreover, it has been demonstrated that the deletion of the M1 mAChR gene increases the receptive fields of V1 neurons [11], i.e., the overlapping areas between neurons thus decrease the efficacy of feed-forward connections due to competition. The post-synaptic effect of M1 may be masked in this study by the nAChR and GABA_A_R facilitatory effects of thalamocortical inputs.

Although there seems to be an enhancement in the non-trained orientation stimulus after VS/HDB training, the effect was not significant. The fact that VEP enhancement is shown only for the trained orientation is in agreement with our previous result showing that an increase of visual discrimination capacity was only observed with trained orientation [3, 26, 27, 71, 72]. The spread to an untrained spatial frequency suggests that the VS/HDB pairing not only improves visual perceptibility by facilitating perceptual learning but can also enhance visual capacity by increasing visual sensitivity.

### Interaction between the cholinergic and GABAergic system

The reductions of the VEPs by M2 mAChR inhibition (VS/AFDX) observed in the present study suggests that M2 mAChRs may act on an inhibitory system, as already suggested by others [12, 19, 20, 73]. This putative role is supported by the anatomical co-localization of M2 mAChRs with GABA neurons [62] and GABAergic terminals [74]. This implies that M2 mAChR blockade can increase GABAergic neuronal activity/release resulting in an increase of intracortical inhibition of the thalamocortical response [70]. However, direct injection of GABAergic agonists with or without cholinergic stimulation (VS/HDB/MUS and VS/MUS) did not significantly block the enhancement effect of VS/HDB stimulation or reduce the VEP amplitude in VS group, respectively. In primates, the proportion of M2 mAChRs on excitatory and inhibitory neurons is similar (<10% vs 6%) [62], so an additive effect of M2 antagonist on the excitatory and inhibitory cells might induce the strong M2 mAChR suppression effect. Excitatory AChRs are also expressed at the cell surface of GABAergic neurons (e.g., nAChR [9, 18]), where they could enhance the GABAergic drive in VS or VS/HDB condition, which could explain the reductions in VEP amplitudes that were observed to be below control levels following the blockade of M2 mAChRs (VS/HDB/AFDX and VS/AFDX) [62].

In summary, ACh released during repetitive VS/HDB stimulation enhances thalamocortical processing through (i) nAChRs, increasing thalamocortical transmission; (ii) M1 mAChRs, resulting in a restriction of lateral spread; and (iii) M2 mAChRs, which may disinhibit pyramidal neurons or spiny stellate cells via the inhibition of GABAergic drive and enhance cortical activity during visual processing. Visual function is amplified by synchronized neuronal activity in the gamma band oscillation that is induced by VS/HDB pairing. The synchronization of cortical microcircuitry requires a fine balance of the action of all of the different classes of receptors.
